# Blood Pressure Variability in Hypertension: A Rehabilitation Perspective

**DOI:** 10.3390/jcdd12080317

**Published:** 2025-08-19

**Authors:** Manikandan Raju, Marco Alfonso Perrone, Anas R. Alashram, Ferdinando Iellamo

**Affiliations:** 1 Department of Neurorehabilitation Sciences, Casa-di-Cura Igea, 20144 Milan, Italy; 2 Department of Human Neuroscience, Sapienza University of Rome, 00185 Rome, Italy; 3 Department of Clinical Sciences and Translational Medicine, University of Rome Tor Vergata, 00133 Rome, Italy; 4 Department of Physiotherapy, Middle East University, Amman 11831, Jordan

**Keywords:** blood pressure variability, hypertension, rehabilitation

## Abstract

The role of blood pressure variability (BPV) as an important marker of cardiovascular (CV) health, specifically its relationship with arterial stiffness and left ventricular remodeling in patients with hypertension, was investigated. This review aimed to elucidate the intricate relationship between BPV, arterial stiffness, and cardiac remodeling. BPV, as both a risk factor and a target of treatment, was also evaluated. The results point to the pivotal role of BPV in cardiovascular events, serving as an independent factor contributing to arterial stiffness and adverse left ventricular remodeling. The article concludes that BPV is a modifiable risk factor and that there is a need for an intervention in specific regions. BPV is a therapy target that is significant in the treatment of hypertension. The optimization of risk and prevention needs a multidisciplinary approach involving rehabilitation therapy, which will improve cardiovascular conditions and patient outcomes.

## 1. Introduction

Blood pressure variability (BPV) is defined as variations in BP over various time intervals, like beat-to-beat over a day or over a few days, and from clinic visit to clinic visit [1,2]. Unlike the traditional mean blood pressure, BPV quantifies dynamic blood pressure variability, which may express features of underlying cardiovascular dysregulation. In hypertension, increased BPV has been associated with adverse cardiovascular outcomes independent of mean blood pressure levels [3]. Additionally, for any 25 h average BP, target organ damage can be severe in patients with higher BP variability. Higher BPV in hypertensive patients has been linked to a higher risk of stroke, coronary heart disease, and mortality, highlighting its prognostic significance regardless of static BP levels [4].

The physiological substrate of BPV is multifactorial and includes interaction between neural, humoral, and mechanical mechanisms that influence vascular tone and cardiac output [2]. Such dynamic variability can cause vascular remodeling, endothelial dysfunction, and myocardial stress and result in structural and functional changes in the heart and vasculature. The identification of early markers of cardiovascular impairment in patients with essential hypertension and high BPV can result in risk stratification and intervention [2,3].

In the cardiovascular system, left ventricle and arterial wall stiffness are critical to cardiac performance and vascular health. Left ventricular stiffness is a diseased state in which the heart muscle, particularly the left ventricle, is less compliant, and its role in filling with blood during diastole is impaired [5]. Similarly, arterial stiffness reflects the rigidity of large arteries that occurs when the elastic function of the arterial wall is compromised more frequently due to aging or chronic hypertension [6]. Increased arterial stiffness is a marker of vascular aging and a predictor of cardiovascular events like myocardial infarction and stroke [7]. Current evidence suggests a connection between BPV and left ventricular and arterial stiffness, in which BP variability may be implicated in vasculature and heart structural and functional changes [8]. However, the precise mechanisms through which BPV is connected with these measures of stiffness are not yet understood. Consequently, the objective of the present review is to find and synthesize current evidence for BPV association with left ventricular and arterial stiffness in hypertensive patients. An exploration of such interactions will provide some insights into the role of BPV in the cardiovascular stiffness pathophysiology and hypothesize its potential utility in hypertension treatment and prognosis.

**Figure 1:**
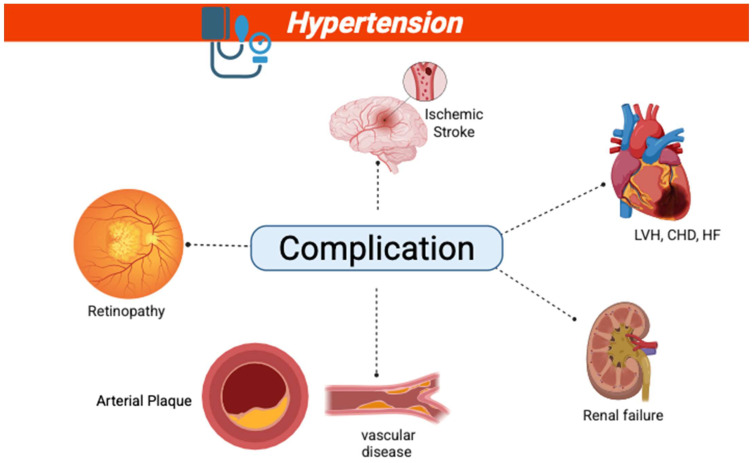
Complications of hypertension: LVH—left ventricular hypertrophy; CHD—coronary heart disease; HF—heart failure.

Some of the complications that could arise due to hypertension are presented in Figure 1.

## 2. Types of Blood Pressure Variability

BPV refers to fluctuations in BP over different time intervals. BPV provides information in addition to mean blood pressure readings that capture dynamic changes that can affect cardiovascular health. BPV is typically classified into different types based on the time interval over which fluctuations take place, each having distinct implications for cardiovascular outcomes [9,10]. Short-term BPV is beat-to-beat and day-to-day variability, like that of 24 h variation. Physical activity, stress, and diurnal rhythms may cause this variation. Short-term BPV is typically measured with the help of ABPM and is associated with cardiovascular morbidity, especially where there is great variability [11]. Long-term BPV encompasses variations lasting weeks, months, or years. Variations can be quantified by comparing clinic BP levels or using multiple ABPM procedures performed over long periods. Long-term BPV estimates an individual’s global BP control stability, and increased long-term BPV has been linked to increased risk of stroke, coronary events, and all-cause mortality [4]. Visit-to-visit BPV is defined by variation in BP levels between consecutive clinical visits and is influenced by disease severity, clinical conditions, and medication adherence. Studies have shown that increased visit-to-visit BPV is an independent and unique predictor of cardiovascular morbidity and mortality [12]. The variability has been prominently noted in the elderly and those with pre-existing cardiovascular disease [13,14,15].

### Classification and Clinical Implications of BPV

1. Short-term BPV
  1. Definition: fluctuations occurring over minutes, hours, or 24 h periods (beat-to-beat or diurnal changes);
  2. Causes: physical activity, stress, autonomic dysfunction, and diurnal rhythms;
  3. Measurement: assessed via ambulatory blood pressure monitoring (ABPM) or continuous BP devices;
  4. Clinical Relevance: Elevated short-term BPV is associated with cardiovascular morbidity, particularly in individuals with high variability. It is also linked to target organ damage, such as left ventricular hypertrophy and arterial stiffness [11].
2. Long-term BPV
  1. Definition: variations spanning weeks, months, or years, reflecting instability in BP control;
  2. Measurement: quantified through repeated clinic visits or serial ABPM over extended periods;
  3. Clinical Relevance: increased long-term BPV predicts stroke, coronary events, and all-cause mortality, especially in hypertensive patients [4].
3. Visit-to-Visit BPV
  1. Definition: variability in BP levels between consecutive clinical visits;
  2. Drivers: disease severity, clinical conditions, medication adherence, and white-coat effect;
  3. Clinical Relevance: increased visit-to-visit BPV is an independent predictor of cardiovascular morbidity and mortality, particularly in the elderly and those with pre-existing cardiovascular disease [12].
4. Normal vs. Altered BPV: Thresholds and Clinical Significance

Normal BPV values have been derived to separate physiological variation from pathological variability that is linked to heightened cardiovascular risk. In short-term BPV, a 24 h systolic SD < about 12 mmHg is normal, indicating stable autonomic and vascular function. Values > 15 mmHg are abnormal and have been shown to be associated with a twofold risk of stroke and target organ damage Parati et al. (2018) [2]. Long-term BPV, quantified over weeks to years, is normally <10 mmHg SD, with values > 12 mmHg SD predictive of stroke risk, coronary events, and all-cause mortality, according to Muntner et al. (2011) [14] and Rothwell et al. (2010) [4]. Visit-to-visit BPV also has normal values of <10 mmHg SD, with greater variability independently related to greater cardiovascular mortality, particularly in the elderly and in those with cardiovascular disease (Muntner et al., 2011 [14]. Establishment of these cut-offs is required for the purposes of initiating timely clinical action since elevated BPV justifies intensified therapy to mitigate target organ damage and cardiovascular risk.

## 3. Mechanisms Affecting Blood Pressure Variability

BPV is caused by complex mechanisms that involve the nervous system, endocrine regulation, and vascular responsiveness. The autonomic nervous system plays a prime role in the regulation of blood pressure variability, particularly due to the interaction between sympathetic and parasympathetic activity. The sympathetic nervous system reacts to stress and stimuli, leading to acute increases in BP. Sympathetic overactivation, commonly present in hypertensive patients, is one of the causes of increased BP variability and could ultimately lead to target organ damage [11,16,17,18,19]. Adrenaline, cortisol, and angiotensin II are a few of the hormones that play roles in regulating BP and thus BPV. The renin–angiotensin–aldosterone system (RAAS), for example, regulates BP through vascular tone control and sodium retention. The dysregulation of such hormonal systems, common in hypertensive patients, might increase BPV [20,21,22]. In addition, the cortisol circadian rhythm, sympathetic nervous system activity, and renin–angiotensin–aldosterone system (RAAS) are also responsible for daily variations in BP that affect early-morning BP peaks. Vascular Mechanisms: Arterial stiffness and endothelial function are the major vascular determinants of BPV. Stiffer arteries have reduced buffering capacity for pressure changes, resulting in increased BP fluctuations [23,24]. Endothelial dysfunction, most often due to inflammation or oxidative stress, can result in damage to vasodilation, further destabilizing blood pressure regulation [25].

## 4. Impact of Blood Pressure Variability on Cardiovascular Structure and Function

### 4.1. Effects on Cardiac and Arterial Structures

Increased BPV has been associated with harmful alterations in cardiac as well as arterial morphology. BPV increase can potentially lead to endothelial dysfunction preceding atherosclerosis by causing intermittent shear stress on vascular endothelium. This tension damages endothelial cell function, which causes inflammation and plaque formation, thus contributing to arterial stiffness and reduced compliance [5,8,25,26]. This stiffness of the arteries, in turn, enhances heart workload since the heart must pump against elevated resistance, with the potential to cause left ventricular hypertrophy (LVH) and remodeling.

### 4.2. Role in Left Ventricular Hypertrophy and Remodeling

BPV contributes to LVH and remodeling through numerous mechanisms. Repeated pressure overload due to the variability of BP brings about the growth of the myocardial cells and fibrosis, contributing to increased left ventricular mass and altered geometry [27,28]. Such remodeling can lead to compromised diastolic function and reduced cardiac efficiency. Studies have proven that greater BPV is independently associated with greater left ventricular mass and greater concentration of concentric hypertrophy when adjusting for mean blood pressure levels [29]. This proves that BPV independently contributes to cardiac structural remodeling apart from its effect of chronic hypertension.

### 4.3. Left Ventricular Stiffness and Blood Pressure Variability

BPV has been found to be independently associated with reduced left ventricular compliance. In both hypertensive and normotensive individuals, the relationship between increased BPV and impaired relaxation of the myocardium and stiff left ventricle has been noted. For instance, according to studies, patients with higher BPV have higher diastolic dysfunction, as evidenced by echocardiographic findings like increased E/e’ ratio and left atrial volume, indicators of left ventricular stiffness and filling pressures [30]. BP fluctuations induced in animal models lead to progressive stiffening of the ventricles, supporting the finding that it is BPV itself, besides the average BP, that is responsible for left ventricular compliance changes.

## 5. Mechanisms Linking BPV to Left Ventricular Hypertrophy

Intermittent Pressure Overload: Repeated BP surges subject the heart to a variable load that triggers compensatory hypertrophy to manage the intermittent stress. The pressure surges deliver a stimulus for myocardial expansion and collagen deposition that increase left ventricular mass and reduce compliance over time [31,32,33]. Myocardial Fibrosis: BPV is also associated with increased myocardial fibrosis, a process with excessive deposition of collagen within the myocardium that interferes with elasticity [34]. Such fibrosis is in part mediated through the activation of RAAS during episodes of increased BP, favoring the synthesis and deposition of collagen [35]. Sympathetic Activation: BPV is often followed by heightened sympathetic nervous system activity, particularly in response to stressors. Sympathetic activation causes cardiomyocyte hypertrophy and can exacerbate the fibrotic response of the myocardium with additional stiffening and dysfunction. Oxidative Stress and Inflammation: BP variability can cause oxidative stress, which participates in the remodeling process. Oxidative stress activates pro-inflammatory pathways that further amplify fibrosis and structural remodeling in the myocardium. This adds to left ventricular stiffening, compounding the adverse effects of BPV on cardiac function [36,37].

## 6. Arterial Stiffness and Blood Pressure Variability

Arterial stiffness is the reduced arterial elasticity that compromises the ability of arteries to dilate and constrict in response to alterations in pressure during the cardiac cycle. It is a marker of vascular aging and an independent predictor of cardiovascular events [8]. The most frequent method of the assessment of arterial stiffness is the measurement of pulse wave velocity (PWV). PWV measures the speed at which pressure waves travel in the arterial tree, with higher values indicating more rigid arteries. Carotid-femoral PWV has become the gold standard of central arterial stiffness and has been shown to have strong predictive value for cardiovascular events and mortality [38,39].

## 7. Arterial Elasticity and Vascular Aging

BPV includes variation in BP over different time intervals, such as beat-to-beat within a day and from visit to visit. Arterial stiffening and vascular aging have been associated with greater BPV. Repeated BP fluctuations place mechanical stress on the arterial wall that could lead to endothelial dysfunction, inflammation, and structural alterations such as increased collagen accumulation and elastin degradation. These changes reduce arterial compliance and accelerate arterial stiffening and result in vascular aging [40,41].

## 8. Clinical Studies Correlating BPV and Arterial Stiffness

A few research works presented a relationship between BPV and arterial stiffness: A study pointed out that higher BPV from visit to visit was associated with higher arterial stiffness measured by PWV in hypertensive patients [26]. There was a relationship between short-term BPV determined by 24 h ambulatory monitoring and arterial stiffness parameters, demonstrating that even short-term variability can negatively affect vascular health [42,43,44,45]. A study concluded that greater BPV is linked with greater arterial stiffness and greater cardiovascular risk, implicating a promising clinical usefulness of BPV control in slowing vascular aging [40].

## 9. Clinical Implications in Hypertension Management

BPV is emerging as an important factor in the management of hypertension because it has been increasingly widely recognized as an independent predictor of cardiovascular morbidity and mortality. BPV factors may affect the treatment strategy in hypertensive patients, especially those at high risk of arterial and ventricular stiffness-related complications. While the traditional approach to treating hypertension is to decrease the mean blood pressure, BPV also provides additional information regarding a patient’s cardiovascular risk. Reducing BPV has been established as equally valuable as decreasing mean blood pressure to minimize adverse effects [4]. The treatment can then be optimized not just to lower total blood pressure but also to normalize BP variability.

Certain antihypertensive medications like ACE inhibitors and calcium channel blockers have been proven to decrease BPV considerably. For instance, the calcium channel blocker amlodipine in once-daily dosage has been proven to decrease BPV more than any other antihypertensive drug class and is particularly helpful in patients with increased BPV. Similarly, ARBs like losartan have been linked to improved BP control and could reduce vascular damage caused by BPV [46]. Treating patients with severe BPV would therefore address the drugs proven active against BPV as well as mean BP, as these would have added general cardiovascular protection.

## 10. Targeting BPV to Reduce Cardiovascular Risks

Increased BPV is the cause of the development of arterial stiffness and left ventricular hypertrophy, both of which pose a higher risk of cardiovascular events. Through BPV, it may be possible to slow down or stop the structural changes in the cardiovascular system that are held accountable for heart failure, stroke, and other complications [47]. Reducing BPV can also increase vascular compliance and reduce myocardial workload, thereby averting the harmful effects of arterial and ventricular stiffness. In patients with high BPV, treatment interventions aimed at stabilizing blood pressure can reduce oxidative stress and vasculature inflammation, protecting against endothelial dysfunction and inappropriately increased collagen deposition [48]. This can translate to reduced arterial stiffness and improved left ventricular compliance, which are critical to long-term cardiac function. Clinical presentation of BPV also extends to individualized treatment approaches where continuous monitoring of BPV can guide therapeutic adjustment. Recent evidence also suggests that non-pharmacological interventions such as lifestyle changes to decrease stress, regular physical activity, and lowering dietary sodium intake can have positive impacts on BPV [49]. A combination of lifestyle and pharmacologic therapy to treat BPV can offer a more comprehensive strategy for lowering cardiovascular risk in hypertensive patients.

## 11. Cardiovascular Rehabilitation and Its Role in Managing BPV and Cardiovascular Stiffness

Cardiovascular rehabilitation (CR) has also been promising in the management of BPV and reduction in arterial and ventricular stiffness with its overall, multifaceted system of exercise, lifestyle modification, and patient education. Intermittent, moderate-intensity aerobic exercise, the focal point of CR, has been associated with reduced BPV through autonomic activity stabilization, reduced sympathetic outflow, and improved endothelial function. Greater endothelial fitness with habitual exercise is also the cause of greater arterial elasticity, and reduced sympathetic activity lowers BP peaks that are accountable for stiffness [50]. In addition, CR interventions also entail stress management, which can reduce psychological stressors responsible for triggering BP changes, further stabilizing blood pressure in the long term. Lifestyle modifications, such as sodium restriction and potassium augmentation, facilitate such impacts by reducing vascular pressure and oxidative load. Through the incorporation of these evidence-based interventions, cardiovascular rehabilitation, in addition to augmenting the stability of BP, also mitigates the changes at the vasculature and myocardium levels to finally improve cardiovascular health as well as counteract long-term danger in hypertensive patients [51].

Exercise has played a central role in the regulation of blood pressure variability (BPV) and blood pressure in hypertensive patients. There is abundant empirical evidence indicating that certain exercises, such as aerobic, resistance, mixed, and even isometric training, are associated with significant decreases in central and ambulatory blood pressure as well as BPV. For example, Lopes et al. [52] found a significant reduction of −2.85 mmHg (*p* = 0.008) in central BP variability following a 12-week aerobic training program, together with raised inflammatory markers and improved cardiorespiratory conditioning. Similarly, Caminiti (2021) [53] observed that aerobic and resistance training had a greater effect than aerobic training alone in reducing 24 h systolic BP variability. The acute effect of exercise was also illustrated further by Matias et al. [54] whose research indicated that there was a reduction of about 2 mmHg in the systolic BP standard deviation from one session of mixed training intensity. Such research explained the beneficial effects of both acute exercise intervention and chronic exercise on vasomotor regulation. Furthermore, the experiments carried out by Heffernan et al. and Araújo et al. [55,56] serve as good evidence for the claim that concurrent resistance with mixed training considerably decreases BPV while improving the health of the cardiovasculature in patients with essential hypertension (Table 1).

## 12. Future Directions and Research Gap

Despite growing evidence regarding the relevance of BPV on cardiovascular risk, significant lacunae remain in understanding its specific role within arterial and ventricular stiffness. Current studies often investigate relationships between BPV and cardiovascular outcomes, but definite mechanisms by which BPV contributes to arterial wall and myocardial stiffness have not been established yet. Specifically, limited information is known about the differential effects of short-term and long-term BPV on structural cardiovascular system alterations [65,66]. In addition, the majority of studies were observational; thus, causal relationships between structural alterations of the heart and vasculature and BPV are challenging to identify. Another limitation exists in population heterogeneity within BPV research. Many of the studies are conducted among specific demographic populations, often omitting young adults or patients with first-stage hypertension. Explaining how BPV affects such groups could reveal insights into the prevention of stiffness-related cardiovascular disease at an early stage. Table 2 highlights the future direction of BPV.

## 13. Interventions Targeting BPV to Reduce Stiffness

BPV management is essential in preventing arterial and ventricular stiffness and, as a consequence, lowering cardiovascular risk. CCBs and ACE inhibitors have proven to be beneficial in the decrease in BPV. It is still essential that more research be performed to find the most effective drugs or combinations of drugs to stabilize blood pressure and lower the risk associated with stiffness. One study by Parati et al. (2024) [67] noted that long-acting CCB, such as amlodipine, effectively controls BPV and subsequent cardiovascular risk. Mean blood pressure levels and BPV must be addressed by researchers in a bid to achieve better cardiovascular prognosis. Inflammatory responses as well as oxidative stress have disproportionate roles to play as BPV-hemmed arterial stiffness determinants. The molecular mechanisms by which oxidative stress and inflammation enhance cardiovascular pathologies were described by Zhazykbayeva et al. (2020), and they hypothesized that they are targets for successful interventions [68]. The findings indicate the effectiveness of combining anti-inflammatory and antioxidant therapy with antihypertensive therapy in increasing the reduction in BPV and its impact on cardiovascular stiffness.

More clinical trials are required to attempt such multicomponent interventions for effectiveness. Non-pharmacological interventions also present areas for investigation that are highly promising. Lifestyle changes, i.e., reduction in stress, exercise, and diet (e.g., sodium limitation), can positively influence BPV and Most studies showed reductions in BPV and/or central and ambulatory BP, with combined or high-intensity programs being more effective than single-mode or short-term interventions [57,58,59,60,61,62,63,64]. Research into the efficacy of these interventions on BPV in and of itself, as well as on arterial and ventricular stiffness, is essential to create wide-ranging, multifaceted strategies for hypertension control. Lastly, advances in technology for monitoring, such as wearables and home-based blood pressure monitors, are an opportunity for the evaluation and management of BPV to become more integrated in everyday practice. Longitudinal research with such technology has the ability to provide useful information on the effectiveness of current BPV control in reducing cardiovascular stiffness and long-term consequences.

## 14. Conclusions

BPV has proven to be a significant actor in hypertension therapy with its role in cardiovascular performance, particularly arterial and ventricular stiffness. This review reveals the mechanism through which BPV generates unwanted structural changes like arterial stiffening, endothelial dysfunction, and left ventricular hypertrophy through pressure overload, inflammation response, and sympathetic stimulation. Augmented BPV has been associated with increased cardiovascular risk independent of mean BP, and this emphasizes the need for control of average BP as well as BP variability. The clarification of the influences of BPVs on cardiovascular stiffness presents a potential area for optimizing the treatment strategy of hypertensive patients. Regulating BPV potentially prevents or delays vascular aging and left ventricular dysfunction and thereby lessens the risk for heart failure, stroke, and myocardial infarction as complications. Regulation of both BP variability and average BP values is essential. Elucidating the function of BPV in cardiovascular stiffness may provide insights for enhancing therapeutic regimens in hypertensive patients. Through BPV intervention, the clinician is able to retard or prevent vascular aging and left ventricular dysfunction, thereby reducing the risk of complicating conditions such as heart failure, stroke, and myocardial infarction. Exercise is vital for regulating BP in people with HT. Various forms of exercise, including aerobic, resistance, mixed, and isometric training, have been shown to reduce both central and ambulatory BP. Studies show that sustained exercise programs have chronic benefits, while acute exercise sessions have immediate positive effects. These findings emphasize the importance of targeted exercise interventions in managing HT to improve vascular function, reduce cardiovascular risk, and improve patient outcomes. Drug and lifestyle interventions that attenuate BPV can improve prognosis in patients with hypertension, emphasizing the importance of a multimodal treatment of such patients.

## Figures and Tables

**Table 1:** Blood pressure variability and exercise.

Scheme	Population	Study Type	Age (Years)	Rehabilitation/Exercise Intervention	BPV Measurement and Results	Main Outcomes	Reference
Susana Lopes et al. (2023)	60/HT	RCT	40–75	12-week aerobic training	↓ Central SBP variability by −2.85 mmHg (*p* = 0.008)	Reduced central SBP	[52]
Giuseppe Caminiti et al. (2021)	55/HT	RCT	≥45	12-week aerobic vs. combined (aerobic + resistance) training	↓ 24 h SBP ARV: Combined: 8.8 to 7.1 mmHg; Aerobic: 8.4 to 7.6 mmHg	Combined training is more effective than aerobic alone in reducing BPV	[53]
Matias et al. (2020)	14/HT	SACT	50 and 70	Acute and 10-week combined training	Acute: ↓ SBP-SD by ~2 mmHg; Chronic: no significant BPV change	Acute sessions reduce BPV; long-term effect on BP but not BPV	[54]
Brandão Rondon et al. (2002)	24/HT	RCT	68.9 ± 1.5	Low-intensity bicycle exercise	↓ Short-term BPV ↓ CO, SV, LVDV	Suggested sex-specific responsiveness to BPV modulation	[57]
Heffernan et al. (2013)	21/HT	RCT	61 ± 1	3 sessions/week, 12 weeks, aerobic or resistance exercise	↓ Central BP	Resistance training alone is effective in BP and BPV control	[55]
Araújo et al. (2022)	2643/HT	Cross-sectional study, Multicenter study	61.6 ± 11.9	10 weeks combined resistance + aerobic	↓ Ambulatory 24 h SBP-SD from 12.3 to 10.7 mmHg	Combined training lowered both BP and variability	[56]
Caminiti et al. (2022)	72/HT	SACT	66.1 ± 12.7	3 sessions/week, 12 weeks, combined exercise	↓ Daytime BPV and 24 h mean SBP	HIIT walking was safe and beneficial for BP control	[58]
Diaz et al. (2012)	14/HT	SACT	NA	3 sessions/week, 24 weeks, aerobic exercise	↓ Systolic BPV (SBPV)	CV, SD, ARV, ASV	[59]
Taylor et al. (2019)	24/HT	RCT	43.8 ± 7.3	3 sessions/week, 4 weeks, isometric exercise	BP significantly reduced (12.4/6.2 to 11.8/5.6 mmHg) (*p* < 0.001)	ARV	[60]
MartinezAguirre-Betolaza et al. (2020)	249/HT	RCT	54.2 ± 7.2 53.1 ± 8.6 54.4 ± 7.2 52.9 ± 8.5 67.6 ± 6.6	MICT HVHIIT LVHIIT/2 session/week for 16 weeks	Improvements in BP and ANS	Combined training lowered both BP and ANS control	[61]
Seidel et al. (2021)	66/HT	RCT	60.7 ± 9.9	5 sessions/week, 12 weeks, aerobic, handgrip	↓ Systolic daytime variability (12.1 ± 2.5 to 10.3 ± 2.8, *p* = 0.04) ↓ Central SBP from 145 ± 15 to 134 ± 19 mmHg (*p* = 0.01)	Combined training lowered both BP and variability	[62]
Batista et al. (2022)	47/HT	SACT	58.0 ± 5.0	3 sessions/week, 12 weeks, Mat Pilates	↓ BP, BPV, and HRV	Pilates training lowered both BP and variability	[63]
Mariano et al. (2020)	13/HT	SACT		30 sessions/week, 10 weeks, combined	BP reductions in hypertensive	Acute sessions reduce BPV; long-term effect on BP but not BPV	[64]

SACT: single-arm clinical trial; RCT: randomized controlled trial; HT: hypertension; NA: none; CV: coefficient of variability; ARV: average real variability; MICT: moderate-intensity continuous training; HVHIIT: high-volume and high-intensity interval training; LVHIIT: low-volume and high-intensity interval training; SBP: systolic blood pressure; BPV: blood pressure variability, BP: blood pressure; CO: cardiac output; LVDV: left ventricular end-diastolic volume; SV: stroke volume; ANS: autonomic nervous system control; HRV: heart rate variability; ↓ = lower values.

**Table 2:** BPV and Rehabilitation highlights.

Category	Highlights
BPV Impact	Elevated BPV increases the risk of stroke, organ damage, and death. Acute exercise reduces short-term BPV, but long-term BPV control is crucial to prevent adverse outcomes.
Rehabilitation Impact	Exercise interventions: aerobic, resistance, combined training, HIIT, isometric exercise, and Pilates consistently lower BPV and improve overall BP control. They also positively affect ANS function and vascular health.
Exercise Type	Combining aerobic and resistance training is more effective in lowering BP than aerobic training alone. Resistance training alone also benefits BP and BP control.
Duration and Frequency	Both acute and sustained rehabilitation programs reduce BPV, but acute exercise provides quicker reductions, while long-term exercise contributes to sustained BP improvements.
Population Benefits	Population benefits are observed in hypertensive populations of all ages, including the elderly and those with pre-existing cardiovascular risk factors. Rehabilitation should be tailored to individual patients.
Future direction	Future Research Directions: Optimal exercise doses for BPV reduction, digital health interventions for sustained BPV control, and the combination of exercise and medication. Mechanisms of BPV Reduction: Investigate the mechanisms underlying exercise-induced BPV reduction and its association with cardiovascular events and mortality.

HIIT; high-volume and high-intensity interval training; BPV: blood pressure variability, BP: blood pressure; ANS: autonomic nervous system control.
